# Heterozygous *Hfe* gene deletion leads to impaired glucose homeostasis, but not liver injury in mice fed a high‐calorie diet

**DOI:** 10.14814/phy2.12837

**Published:** 2016-06-28

**Authors:** Laurence Britton, Lesley Jaskowski, Kim Bridle, Nishreen Santrampurwala, Janske Reiling, Nick Musgrave, V. Nathan Subramaniam, Darrell Crawford

**Affiliations:** ^1^ Gallipoli Medical Research InstituteGreenslopes Private HospitalGreenslopesQueenslandAustralia; ^2^ The School of MedicineUniversity of QueenslandHerstonQueenslandAustralia; ^3^ The Department of GastroenterologyPrincess Alexandra HospitalWoolloongabba, QueenslandAustralia; ^4^ QIMR Berghofer Medical Research InstituteHerstonQueenslandAustralia; ^5^ Department of SurgeryNUTRIM School of Nutrition and Translational Research in MetabolismMaastricht UniversityMaastrichtThe Netherlands; ^6^ Sullivan and Nicolaides PathologyGreenslopes Private HospitalGreenslopesQueenslandAustralia

**Keywords:** Diabetes, iron, nonalcoholic fatty liver disease, steatohepatitis

## Abstract

Heterozygous mutations of the *Hfe* gene have been proposed as cofactors in the development and progression of nonalcoholic fatty liver disease (NAFLD). Homozygous *Hfe* deletion previously has been shown to lead to dysregulated hepatic lipid metabolism and accentuated liver injury in a dietary mouse model of NAFLD. We sought to establish whether heterozygous deletion of *Hfe* is sufficient to promote liver injury when mice are exposed to a high‐calorie diet (HCD). Eight‐week‐old wild‐type and *Hfe* ^+/−^ mice received 8 weeks of a control diet or HCD. Liver histology and pathways of lipid and iron metabolism were analyzed. Liver histology demonstrated that mice fed a HCD had increased NAFLD activity score (NAS), steatosis, and hepatocyte ballooning. However, liver injury was unaffected by *Hfe* genotype. Hepatic iron concentration (HIC) was increased in *Hfe* ^+/−^ mice of both dietary groups. HCD resulted in a hepcidin‐independent reduction in HIC. *Hfe* ^+/−^ mice demonstrated raised fasting serum glucose concentrations and HOMA‐IR score, despite unaltered serum adiponectin concentrations. Downstream regulators of hepatic de novo lipogenesis (pAKT, SREBP‐1, *Fas*,* Scd1*) and fatty acid oxidation (*AdipoR2*,* Pparα*,* Cpt1*) were largely unaffected by genotype. In summary, heterozygous *Hfe* gene deletion is associated with impaired iron and glucose metabolism. However, unlike homozygous *Hfe* deletion, heterozygous gene deletion did not affect lipid metabolism pathways or liver injury in this model.

## Introduction

Nonalcoholic fatty liver disease (NAFLD) is increasingly common in the developed and developing world, affecting around 30% of many adult populations (Amarapurkar et al. 2007; Vernon et al. 2011). The advanced form of the disease, nonalcoholic steatohepatitis (NASH), can lead to life‐threatening complications including liver failure and liver cancer (Anstee et al. 2013). At present, effective treatment strategies to halt or reverse the natural history of NASH are lacking. Cofactors such as type II diabetes mellitus and iron overload have been implicated in NASH pathogenesis and represent readily treatable therapeutic targets (Dongiovanni et al. 2011; Smith and Adams 2011). A greater understanding of the mechanisms by which such cofactors promote NASH disease progression is essential in order to develop effective treatments.

**Figure 1:**
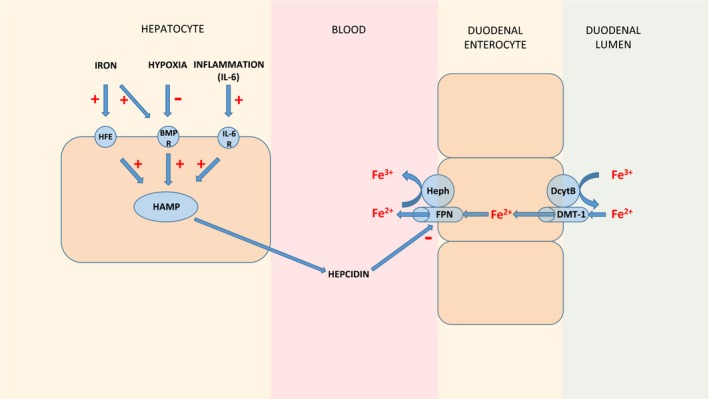
Overview of the regulation of mammalian iron homeostasis. The regulation of mammalian iron homeostasis is predominantly achieved via the control of duodenal absorption of iron by the hormone hepcidin. Hepcidin causes the internalization and degradation of the iron‐exporter ferroportin, thus reducing the passage of iron from enterocytes into the circulation. Other key proteins involved in duodenal iron absorption, divalent metal transporter 1 (DMT‐1), and the two oxidoreductases, hephaestin (Heph) and duodenal cytochrome B (DcytB), are shown. The *HAMP* gene encodes hepcidin and is regulated by a number of factors including iron, via the HFE and bone morphogenetic protein 6 (BMP‐6) pathway, hypoxia, via the BMP‐6 pathway, and inflammation, via an interleukin‐6 receptor (IL‐6) pathway. R denotes receptor. Adapted from Fleming et al. (Fleming and Ponka 2012) and Chua et al. (Chua et al. 2007).

Iron overload due to homozygous p.C282Y mutation of *Hfe* is responsible for the majority of cases of hereditary hemochromatosis seen worldwide (Bacon et al. 2011). Heterozygous p.C282Y mutations are found in approximately 11% of Caucasian populations and are associated with increased iron stores, but not with liver disease in the absence of an additional cofactor (Allen et al. 2008). In a large meta‐analysis, *Hfe* gene mutations have been shown to convey an increased risk of nonalcoholic steatohepatitis (Ellervik et al. 2007). Among individuals with NAFLD, the presence of the heterozygous p.H63D mutation of *Hfe* has been shown to be associated with more advanced histological injury as assessed by the NAFLD activity score (Nelson et al. 2012). An overview of the role of HFE in mammalian iron metabolism is summarized in Figure 1.

Liver injury in both hemochromatosis and NASH is characterized by the presence of oxidative stress (Bacon et al. 2011; Rolo et al. 2012). Insulin resistance, itself associated with oxidative stress, is commonly observed both in individuals with NASH and also in those with hemochromatosis (Smith and Adams 2011; Simcox and McClain 2013). Given the prevalence of *Hfe* gene mutations and shared pathogenic mechanisms with NASH, they have received intense interest in recent years as potential cofactors in NAFLD disease progression.

Previous work from our group has demonstrated that mice with homozygous *Hfe* deletion which are fed a high‐calorie diet (HCD) develop steatohepatitis and early fibrosis (Tan et al. 2011). This effect of diet is not seen in wild‐type controls which develop only simple steatosis. In this model, *Hfe* null mice demonstrated upregulation of gene expression of de novo lipogenesis pathways and downregulation of fatty acid oxidation pathways. This imbalance of fatty acid synthesis and oxidation may explain the liver injury seen in these *Hfe* null mice. However, the mechanisms by which *Hfe* deletion might dysregulate hepatic lipid metabolism remain to be defined.

These findings led us to consider whether a partial deficiency of functional HFE, as seen in those with heterozygous C282Y and H63D mutations, might be sufficient to dysregulate hepatic lipid metabolism and promote liver injury in NAFLD. In this article, we sought to explore the mechanisms of interaction between heterozygous *Hfe* gene mutations and nonalcoholic fatty liver disease. We hypothesized that heterozygous *Hfe* deletion promotes dysregulated hepatic lipid metabolism as seen in the homozygous model. To test this hypothesis, we applied the 8‐week high‐calorie diet model of NAFLD, previously used in *Hfe* homozygous null mice, to mice with and without heterozygous deletion of *Hfe*.

## Materials and Methods

### Experimental animals

Eight‐week‐old male C57BL6/J *Hfe* ^+/−^ mice (bred at the QIMR Berghofer Medical Research Institute, Brisbane, Australia) and wild‐type (WT) littermate controls were assigned to receive a control diet (CD) or a high‐calorie (40.5% sucrose, 23.5% fat, 0.19% cholesterol by weight) diet (HCD) for 8 weeks (*n* = 10 per group). The constituents of these diets are summarized in Table 1. Both diets contained 1.3 *μ*mol/g iron. The high‐calorie diet is analogous to a “Western” style diet, containing a high content of fat, simple carbohydrates, and cholesterol (Tan et al. 2011). Both diets were supplied by Specialty Feeds, Glen Forrest, Western Australia. All animals were cared for in accordance with the NHMRC code for the care and use of animals for scientific purposes and with approval of the Animal Ethics Committee of the QIMR Berghofer Medical Research Institute. Mice were housed in a temperature controlled environment (23°C) with a 12:12‐h light:dark cycle. Mice had ad libitum access to diet and water.

**Table 1:** Major components of experimental diets

Dietary component	Control diet	High‐calorie diet
Protein (% weight)	19.4%	19.4%
Total fat (% weight)	7.0%	23.0%
Total carbohydrate (% weight)	61.7%	50%
Digestible energy (MJ/kg)	16.1	20
Cholesterol (% weight)	0%	0.19%
Casein (acid) (g/kg)	200	200
Sucrose (g/kg)	100	405
Canola oil (g/kg)	70	50
Cellulose (g/kg)	50	50
Wheat starch (g/kg)	404	50
Dextrinized starch (g/kg)	132	0
Cocoa butter (g/kg)	0	50
Hydrogenated vegetable oil (g/kg)	0	131

Sixteen‐week‐old mice were sacrificed by general anesthesia with intraperitoneal injection of ketamine and xylazine, following a 5‐h fast. Blood was collected by cardiac puncture and serum was stored at −80°C. Livers were excised, and pieces of tissue were either dried at 100°C for 72 h for hepatic iron concentration determination, fixed in formalin for histology, or snap frozen in liquid nitrogen and stored at −80°C. Small bowel enterocytes were collected as described previously (Chen et al. 2003; Fuqua et al. 2014). In brief, 10‐cm sections of proximal small bowel were cut longitudinally then washed in ice‐cold phosphate‐buffered saline (PBS). Samples were then gently rotated for 30 min at 4°C in 1.5 mmol/L ethylenediaminetetraacetic acid (EDTA) in PBS. The gut tissue was removed and the remaining sample containing enterocytes was centrifuged at 500 *g* to pellet the enterocytes.

### Histological assessment

Formalin‐fixed samples were embedded in paraffin. Sections were stained with hematoxylin and eosin (H&E) for the assessment of liver injury. Sirius red staining was used to detect the presence of hepatic fibrosis. Histological scoring was performed by an expert histopathologist blinded to the study groups according to criteria described by Kleiner et al. (Kleiner et al. 2005).

### Hepatic iron concentration

Oven dried liver samples weighing 4–9 mg were added to 300 *μ*L of concentrated nitric acid. Duplicate samples for each liver were then digested in a heated sand bath. Chromogen reagent was prepared (0.1% banthophenanthroline sulfate, 1% mercaptoacetic acid). One part of chromogen reagent was added to five parts of saturated sodium acetate to make working chromogen reagent immediately prior to use. When fully digested, the volume of each sample was determined by pipette and 25 *μ*L was added to 125 *μ*L working chromogen reagent. Absorbance was measured at 540 nm on a plate reader, Tecan infinite F200, Tecan, Switzerland. The iron concentration by dry weight was determined with reference to an iron standard (Iron standard for AAS, Sigma, St. Louis).

### Serum analysis

Serum alanine aminotransferase (ALT) was measured on a Beckman (DxC800) General Chemistry Automated Analyser (Beckman Coulter, Fullerton). Serum glucose and insulin were measured on a Cobas Integra 400 Chemistry Automated Analyser (Roche Diagnostics, Basel, Switzerland).

### RNA extraction and real‐time quantitative PCR (RT‐qPCR)

RNA was extracted from tissue homogenates using QIAZOL reagent (Qiagen, Hilden, Germany). After treatment with deoxyribonuclease 1, cDNA was synthesized from 1 *μ*g RNA, using superscript III reverse transcriptase (Invitrogen, Mulgrave, Australia). RT‐qPCR was performed in a ViiA 7 real‐time PCR machine (Invitrogen) with a Quantifast SYBR Green master mix (Qiagen) and thermal cycling as follows: 95°C for 5 min then 40 cycles at 95°C for 10 sec followed by 60°C for 30 sec prior to a melt curve analysis for validation. Relative mRNA expression was determined by calibration of Ct values to a standard curve of dilutions of a pooled mix of cDNA samples. Expression was then normalized to geometric mean of three reference genes glyceraldehyde‐3‐phosphate dehydrogenase (*Gapdh*), basic transcription factor‐3 (*Btf3*), and beta‐2‐microglobulin (*B2‐mg*). Primer sequences that were used are shown in Table 2.

**Table 2:** RT‐qPCR primer sequences (5′ to 3′)

	Forward primer	Reverse primer
*Hamp1*	TTGCGATACCAATGCAGAAG	GGATGTGGCTCTAGGCTATGTT
*Dmt1*	CCAGCCAGTAAGTTCAAGGATC	GCGTAGCAGCTGATCTGGG
*Hephaestin*	CCGACCTTACACCATTCACC	GGACAGAATCATCCGCTTTC
*Fas*	TACCAAGCCAAGCACATTCG	TGGCTTCGGCATGAGA
*AdipoR2*	TACACACAGAGACGGGCAAC	TGGCTCCCAAGAAGAACAAG
*Pparα*	CATGTGAAGGCTGTAAGGGCTT	TCTTGCAGCTCCGATCACACT
*Cpt1*	AGACCGTGAGGAACTCAAACCTA	TGAAGAGTCGCTCCCACT
*Gapdh*	TCCTGCACCACCAACTGCTTAGC	GCCTGCTTCACCACCTTCTTGAT
*Btf3*	TGGCAGCAAACACCTTCACC	AGCTTCAGCCAGTCTCCTTAAAC
*B2‐mg*	CTGATACATACGCCTGCAGAGTTAA	ATGAATCTTCAGAGCATCATGAT

### Western blotting

Serum adiponectin levels were determined by western blotting. Samples of 1:1000 serum (5 *μ*L) were electrophoresed in 2% Agarose (in Tris‐Glycine) gels (Lonza, Basel, Switzerland) at 70 V for 60 min. Protein levels of phosphoAKT (pAKT) and sterol regulatory element binding protein‐1 (SREBP‐1) were determined using western blotting of liver tissue extracts. Protein concentration was quantified using a Pierce BCA Protein Assay Kit (Thermo Scientific, Rockford). Thirty micrograms of protein from whole liver protein extracts (pAKT) and 10 *μ*g of protein from liver nuclear extracts (SREBP‐1) were electrophoresed in 10% sodium dodecyl sulfate–10% polyacrylamide gels for 10 min at 75 V, then 50 min at 150 V. Samples were then transferred onto polyvinylidene fluoride (PVDF) membranes (BioRad, Hercules) at 100 V for 60 min. Membranes were blocked in 5% skim milk before immunostaining with primary antibodies. Secondary antibody binding was performed using horseradish peroxidase (HRP) antibodies. Visualization was performed using a standard chemiluminescent kit (Supersignal West Femto, Thermo Scientific, Waltham) on a 4000MM pro Image Station (Carestream Health, Inc., New York). pAKT and SREBP‐1 band net intensity were normalized to the reference proteins GAPDH and Histone‐H3, respectively. Primary and secondary antibodies used for western blots were as follows: adiponectin (MAB3608, Millipore) 1:10,000, goat anti‐mouse HRP (Invitrogen) 1:200,000; pAKT (sc‐7985‐R, Santa Cruz) 1:1000, goat anti‐rabbit HRP (Invitrogen) 1:200,000; SREBP‐1 (sc‐367, Santa Cruz) 1:500, goat anti‐rabbit HRP (Invitrogen) 1:100,000; GAPDH (MAB374, Millipore) 1:150,000, goat anti‐mouse HRP (Invitrogen) 1:100,000; histone‐H3 (FL‐136, sc10809, Santa Cruz) 1:100, goat anti‐rabbit HRP (Invitrogen) 1:6000. All samples were processed concurrently using three gels with a minimum of three samples per group on each gel.

### Statistical analysis

Statistical analysis was performed using GraphPad Prism 6 software (GraphPad, San Diego, CA). For normally distributed continuous data, groups were compared using two‐way ANOVA based on diet and genotype. For instances in which a significant interaction existed between diet and genotype, two predefined post hoc comparisons to evaluate the effect of genotype for each diet were performed using Sidak's multiple comparisons test. These comparisons were (2008) WT CD versus *Hfe* ^+/−^ CD and (2007) WT HCD versus *Hfe* ^+/−^ HCD. In cases in which a significant interaction did not occur, *P* values relating to overall effect by two‐way ANOVA of diet and genotype are reported. As initial body weight was measured prior to dietary intervention, an analysis between the two genotypes (*n *=* *20 per group) was performed using an unpaired Student's *t*‐test.

For continuous data that were not normally distributed, a Kruskal–Wallis test was performed. When the results of this test were significant (*P *<* *0.05), post hoc comparisons to evaluate the effect of genotype were performed using Dunn's multiple comparisons test (group comparisons as for Sidak's, above). Data for continuous variables are represented graphically with box and whisker plots, demonstrating the maximum, minimum, 25th and 75th centile and median values. Categorical data were analyzed by Fisher's exact test and represented in tabular form.

## Results

### Weight gain was largely comparable between genotypes

**Figure 2:**
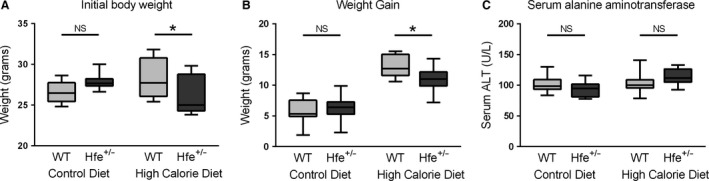
Body weight and serum ALT. (A) Initial body weight (8 weeks). An interaction between diet and genotype was present (*P* = 0.0044, two‐way ANOVA). Post hoc analysis found increased weight in WT mice fed a HCD compared to *Hfe* ^+/−^ mice (*P* = 0.016, Sidak's multiple comparisons test). No significant changes were seen in CD mice. (B) Weight gain (8–16 weeks). An interaction between diet and genotype was present (*P* = 0.040, two‐way ANOVA). Post hoc analysis found increased weight gain in WT mice fed a HCD compared to *Hfe* ^+/−^ mice (*P* = 0.040, Sidak's multiple comparisons test). No significant changes were seen in CD mice. (C) Serum alanine aminotransferase (ALT) is not influenced by diet or genotype. A significant interaction by two‐way ANOVA was present (*P* = 0.047). However, differences between genotype (Sidak's multiple comparisons test) were not significant for either mice fed control diet or HCD (*n* = 9–10 per group).

Weight gain between 8 and 16 weeks of age was largely comparable between genotypes except for a small increase in WT HCD‐fed mice compared to their *Hfe* ^+/−^ counterparts (13.1 g vs 11.0 g, *P* = 0.04, Sidak's multiple comparison test after two way ANOVA). This effect may be related to a higher initial weight at 8 weeks in WT mice. (28.4 g vs. 26.0 g, *P *=* *0.016). The higher initial weight is unlikely to be a true genotype effect as a difference was not seen between the two CD groups. All four groups of mice (CD and HCD) had been fed only CD up until this stage. Mean initial weight for all WT mice (*n* = 20) was 27.5 g, compared to 26.9 g for *Hfe* ^+/−^ mice (*n* = 20), *P *=* *0.39, Student's *t*‐test (Fig. 2A and B).

### Diet, but not *Hfe* genotype influences liver injury in this model

**Figure 3:**
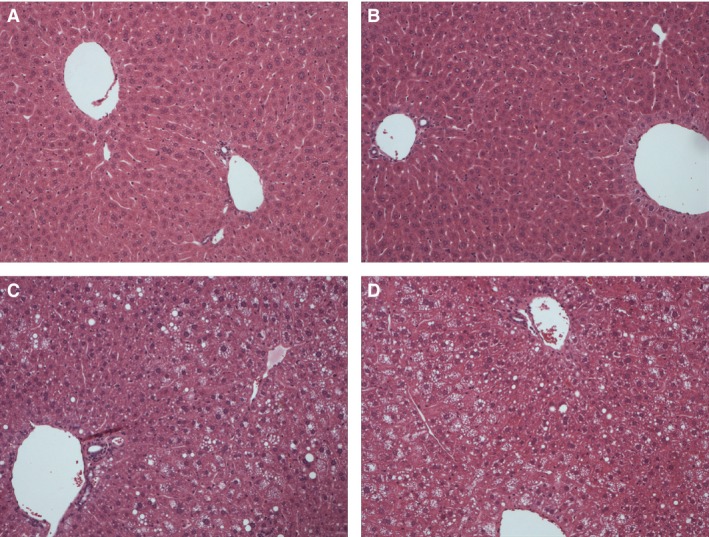
High‐calorie diet leads to increased hepatic steatosis and ballooning degeneration in *Hfe* ^+/−^ mice. Light microscopy of representative liver sections stained with hematoxylin and eosin are shown (original magnification ×100). (A) WT CD. (B) *Hfe* ^+/−^ CD. (C) WT HCD. (D) *Hfe* ^+/−^ HCD.

There was no observed genotype effect on serum ALT (Fig. 2C). Mice fed HCD of both genotypes developed steatosis without overt steatohepatitis (Fig. 3). Table 3 shows the histological scoring of liver sections. NAFLD activity score (NAS) (*P* = 0.003), steatosis (*P* < 0.001), and hepatocyte ballooning (*P* = 0.038) were associated with experimental group by Fisher's exact test. These associations evidently relate to diet rather than genotype. No more than minimal fibrosis was seen in any of the groups (data not shown).

**Table 3:** High‐calorie diet, but not heterozygous *Hfe* gene deletion leads to increased hepatic steatosis, ballooning degeneration, and NAS score

	Control diet		High calorie diet		*P*‐value
	WT	*Hfe* ^+/−^	WT	*Hfe* ^+/−^	
NAS (≥2)	0 (0%)	0 (0%)	5 (50%)	5 (50%)	0.003
Steatosis (Yes)	0 (0%)	0 (0%)	8 (80%)	8 (80%)	<0.001
Lobular Inflammation (Yes)	4 (40%)	3 (30%)	6 (60%)	6 (60%)	0.54
Ballooning (Yes)	0 (0%)	0 (0%)	4 (40%)	2 (20%)	0.038

Number of mice (%) with NAS (≥2), any macrovesicular steatosis (≥ grade 1), lobular inflammation (≥ grade 1), or ballooning (≥ grade 1). *P*‐value is the result of Fisher's exact test. (*n* = 10 per group).

### Hepatic iron concentration (HIC) is increased in *Hfe* ^+/−^ mice in both dietary groups, consistent with the expected phenotype

**Figure 4:**
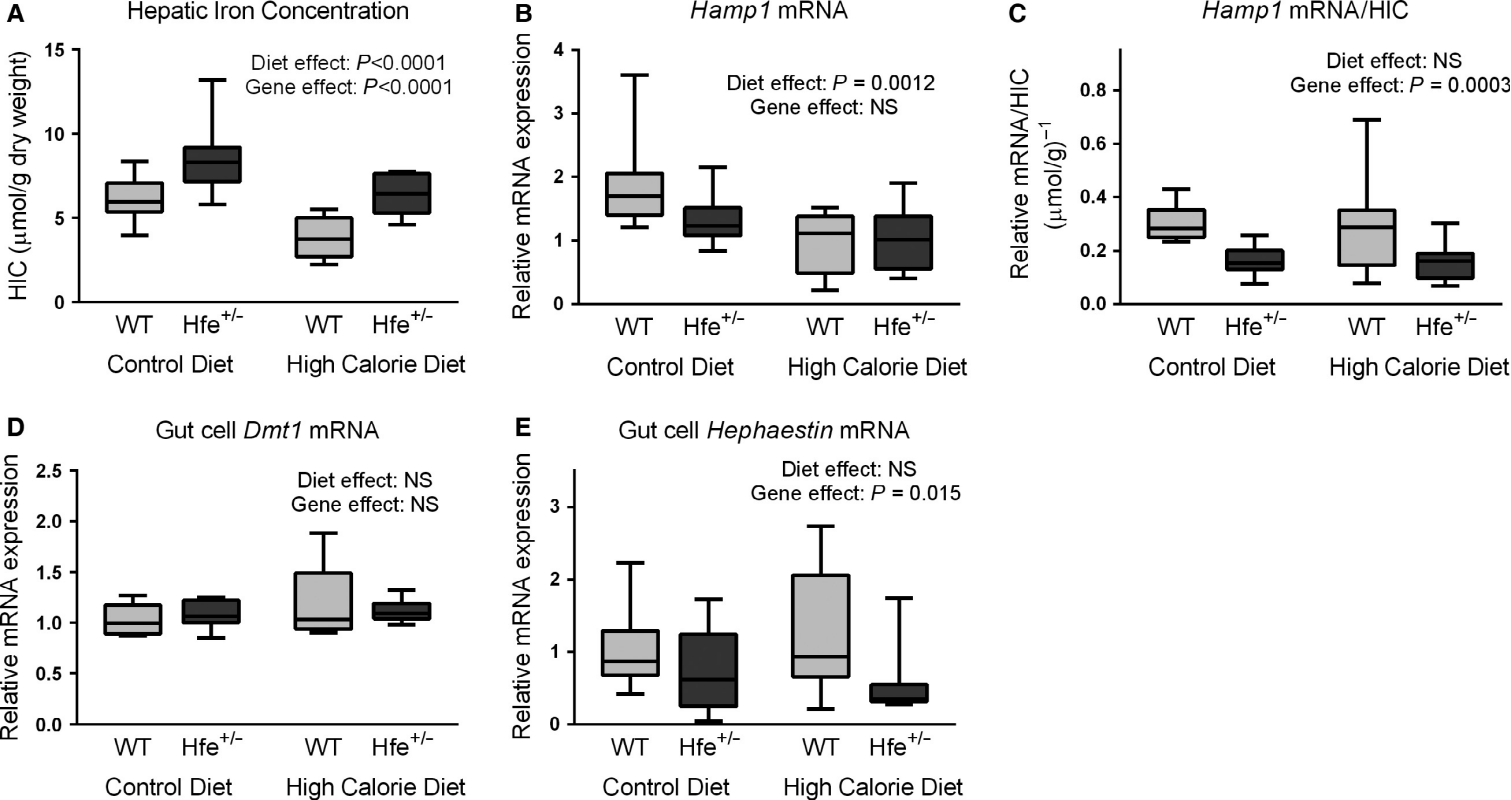
High‐calorie diet‐induced reduction in hepatic iron concentration (HIC) occurs independently of hepcidin. (A) Hepatic iron concentration. HCD was associated with reduced HIC (*P* < 0.0001, two‐way ANOVA). *Hfe* ^+/−^ mice had increased HIC compared to WT mice (*P* < 0.0001, two‐way ANOVA). (B) Hepatic *Hamp1* mRNA expression. *Hamp1* was lower in animals fed HCD (*P* = 0.0012, two‐way ANOVA). Gene effect was not significant (NS). (C) Hepatic *Hamp1* mRNA/HIC. *Hfe* ^+/−^ mice had lower *Hamp1*/HIC ratios (*P* = 0.0003, two‐way ANOVA). Diet effect was NS. (D) Gut cell *Dmt1* mRNA expression. Diet and gene effects were both NS (two‐way ANOVA). (E) Gut cell *Hephaestin* mRNA expression. *Hfe* ^+/−^ mice had lower *Hephaestin* expression than WT mice (*P* = 0.015). Diet effect was NS (two‐way ANOVA). (*n* = 6–10 per group).

*Hamp1* is the gene encoding hepcidin, the master regulator of iron homeostasis (Fig. 4A). When *Hamp1* is expressed as a ratio of HIC, expression was found to be approximately 50% lower in *Hfe* ^+/−^ mice across both dietary groups (Fig. 4C). This observation is consistent with haploinsufficiency of the HFE protein in *Hfe* ^+/−^ mice and supports HFE being the predominant regulator of *Hamp1* expression in this model.

### High‐calorie diet‐induced reduction of HIC occurs independently of hepcidin

HIC was significantly reduced by HCD in both genotypes (*P* < 0.0001, two‐way ANOVA) (Fig. 4A). The explanation for this is unclear. *Hamp1* mRNA expression was lower in HCD‐fed mice (diet effect *P* = 0.0012, two‐way ANOVA, Fig. 4B). The *Hamp1*/HIC results demonstrate that *Hamp1* expression when normalized to HIC is unaffected by diet (Fig. 4C). As the hepcidin–ferroportin axis did not appear to be the cause of the reduced HIC, we sought to evaluate two further regulators of intestinal iron absorption, the divalent metal transporter‐1 (DMT1) and hephaestin. There was no significant diet effect at the mRNA level for either of these by two‐way ANOVA (Fig. 4D and E). *Hfe* ^+/−^ mice demonstrated a reduction in hephaestin mRNA levels. Given the increased iron loading in these mice, this response is consistent with previous work which has shown hephaestin mRNA levels to be regulated by iron (Anderson et al. 2002).

### *Hfe* ^+/−^ mice display impaired glucose homeostasis

**Figure 5:**
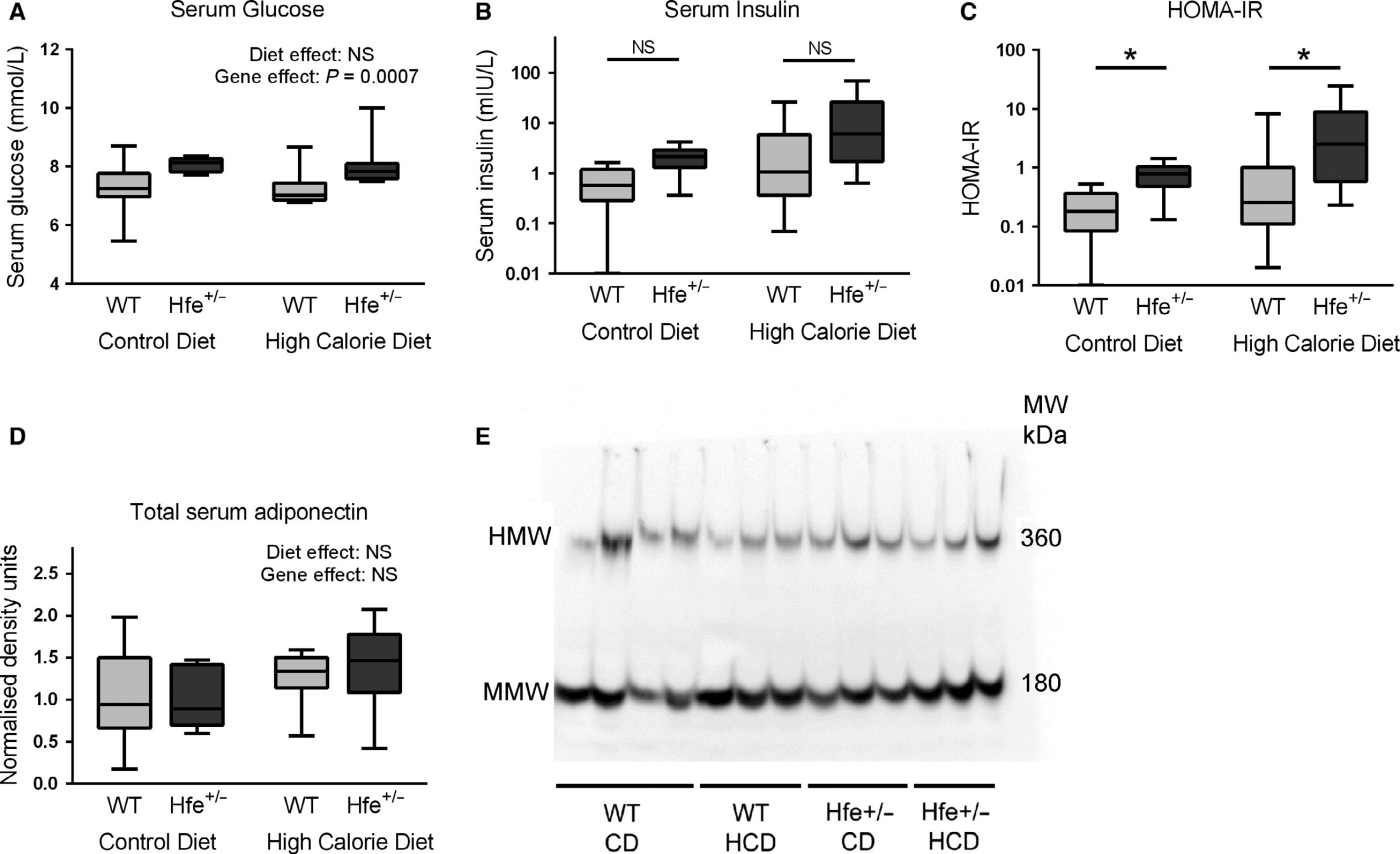
*Hfe* ^+/−^ mice have impaired glucose homeostasis irrespective of diet. (A) Serum glucose. Serum glucose was higher in *Hfe* ^+/−^ mice compared to WT mice (*P* = 0.0007), diet effect was NS (two‐way ANOVA). (B) Serum insulin (mIU/L), presented on logarithmic (base 10) scale. A difference between groups was present *P* = 0.004 (Kruskal–Wallis test). Post hoc comparisons by genotype for CD and HCD mice were both NS (*P* = 0.057, *P* = 0.084, respectively, by Dunn's multiple comparison test). (C) HOMA‐IR, presented on logarithmic (base 10) scale. A difference between groups was present (*P* = 0.0024) (Kruskal–Wallis test). *Hfe* ^+/−^ was associated with increased HOMA‐IR score for mice fed CD (*P* = 0.038) and HCD (*P* = 0.039) (Dunn's multiple comparison test). Immunoblotting for total serum adiponectin, (D) densitometry, (E) representative blots. HMW, high‐molecular‐weight adiponectin. MMW, medium molecular weight adiponectin. Diet and gene effects were both NS (two‐way ANOVA). (*n* = 9–10 per group).

As glucose and insulin are established drivers of hepatic de novo lipogenesis (Leavens and Birnbaum 2011), we measured their fasting serum concentrations. Serum glucose was significantly higher in *Hfe* ^+/−^ than WT animals (genotype effect *P* = 0.0007, two‐way ANOVA); 8.1 mmol/L versus 7.3 mmol/L in CD‐fed mice and 8.0 mmol/L versus 7.2 mmol/L in HCD‐fed mice (Fig. 5A). A trend toward a similar effect was seen for serum insulin although this did not reach statistical significance (Fig. 5B). HOMA‐IR score, which is the product of serum glucose, serum insulin, and a constant, has been shown to be a useful static measure of insulin resistance in the fasting state (Matthews et al. 1985). HOMA‐IR was significantly higher in *Hfe* ^+/−^ mice fed both CD (*P* = 0.038) and HCD (*P*= 0.039) using Dunn's multiple comparison test after a significant Kruskal–Wallis test (*P* = 0.0024) (Fig. 5C). Serum adiponectin has previously been shown to be regulated by iron in mice and is a known key determinant of insulin resistance (Gabrielsen et al. 2012). However, we did not find any significant effect of genotype on total serum adiponectin (Fig. 5D and E). Moreover, when we looked specifically at the active form, high‐molecular‐weight (HMW) adiponectin, we again found no genotype effect irrespective of whether we analyzed absolute HMW adiponectin serum concentration or HMW adiponectin as a fraction of total adiponectin.

### Despite increased serum glucose and HOMA‐IR score, downstream regulators of de novo lipogenesis and fatty acid oxidation are largely unaffected by heterozygous *Hfe* deletion

**Figure 6:**
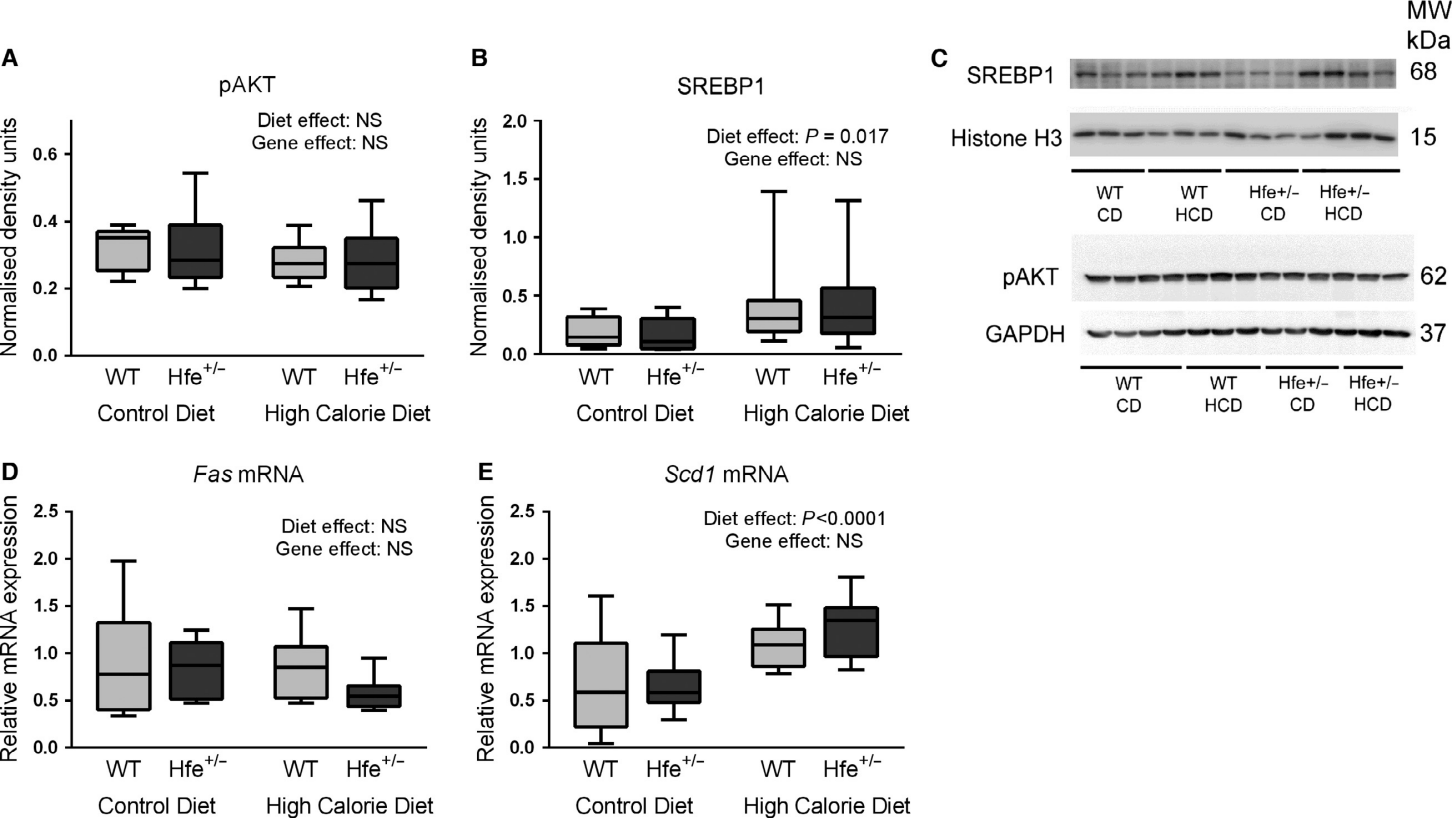
Hepatic de novo lipogenesis pathways are not upregulated despite hyperglycemia. (A) Immunoblotting densitometry of pAKT (whole liver protein extracts) normalized to GAPDH. Diet and genotype effects were both NS (two‐way ANOVA). (B) Immunoblotting densitometry of SREBP‐1 (nuclear protein extracts) normalized to Histone‐H3. HCD was associated with increased nuclear SREBP‐1 (*P* = 0.017). Gene effect was NS (two‐way ANOVA). (C) Representative immunoblots for pAKT, GAPDH, SREBP‐1, Histone‐H3. (*n* = 9–10 per group). (D) Hepatic fatty acid synthase (*Fas*) mRNA expression. Diet and genotype effects were both NS (two‐way ANOVA). (E) Hepatic stearoyl CoA desaturase‐1 (*Scd1*) mRNA expression. HCD was associated with increased *Scd1* expression. Gene effect was NS (two‐way ANOVA).

**Figure 7:**
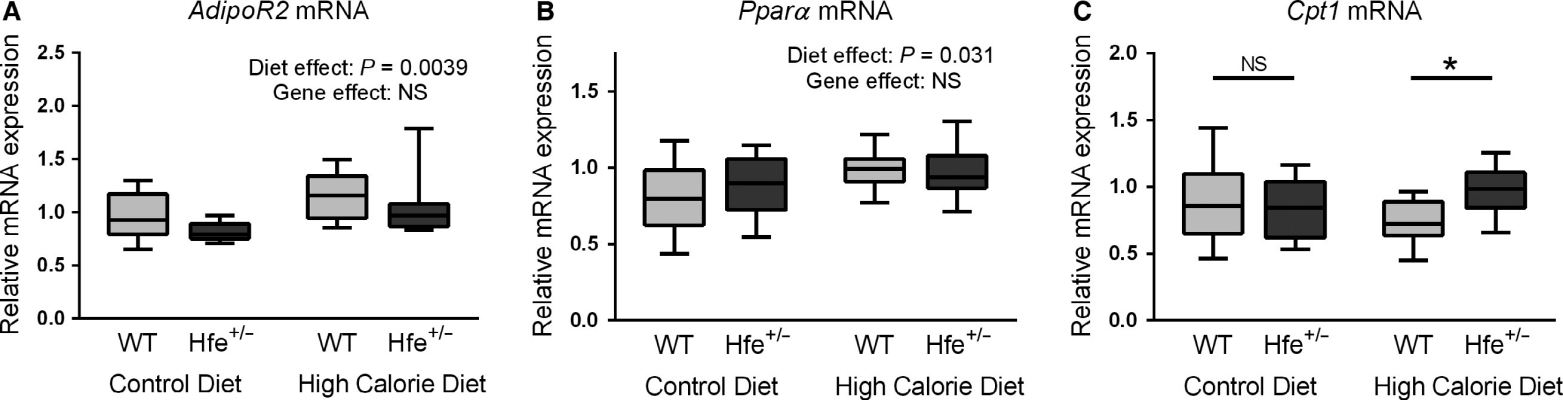
Hepatic lipid metabolism mRNA. (A) Hepatic adiponectin receptor‐2 (*Adipo R2*) mRNA expression. HCD was associated with increased *AdipoR2* expression (*P* = 0.0039). Gene effect was NS (two‐way ANOVA). (B) Hepatic peroxisome proliferator‐activated receptor alpha (*Pparα*) mRNA expression. *Pparα* expression was increased by HCD. Gene effect was NS. (C) Hepatic carnitine palmitoyl transferase‐1 (*Cpt1*) mRNA expression. There was a significant interaction between diet and genotype (*P* = 0.045). Post hoc analysis found increased *Cpt1* expression due to *Hfe* ^+/−^ in HCD‐fed mice (*P* = 0.040). In CD‐fed mice the results are NS (Sidak's multiple comparisons test) (*n* = 10 per group).

The active form of serine/threonine kinase AKT, phospho AKT, which is a central regulator of insulin signaling to lipogenesis pathways, was entirely unaltered by diet or genotype (Fig. 6A and C). Similarly, nuclear extract quantities of sterol regulatory element binding protein‐1 (SREBP‐1), the main transcription factor responsible for regulation of de novo lipogenesis, were also unaffected by genotype (Fig. 6B and C). HCD, however, was associated with an increase in nuclear SREBP‐1 protein, which may be substrate driven. mRNA quantities of fatty acid synthase (*Fas*) and stearoyl CoA desaturase‐ 1 (*Scd1*), which are enzymes involved in hepatic lipogenesis and are downstream targets of SREBP‐1, were also unaffected by genotype (Fig. 6D and E). *Scd1* mRNA, however, was upregulated by HCD, consistent with the observation that *Scd1* is transcriptionally regulated by SREBP‐1 (Mauvoisin and Mounier 2011). Given the lack of genotype effect on liver histology or SREBP‐1 protein levels and that SREBP‐1 in known to act on *Fas* and *Scd1* by regulation of transcription, we focussed on mRNA rather than protein levels of these enzymes (Latasa et al. 2003; Mauvoisin and Mounier 2011). Similarly, there was no effect of *Hfe* heterozygosity on mRNA quantities of three key regulators of fatty acid oxidation: adiponectin receptor‐2 (*Adipo R2*), peroxisome proliferator‐activated receptor alpha (*Pparα*), and carnitine palmitoyl transferase‐1 (*Cpt1*), except for a small increase in *Cpt1* expression in *Hfe* ^+/−^ mice fed a HCD (*P* = 0.040, Sidak's multiple comparisons test) (Fig. 7A–C).

## Discussion

In this study, we have demonstrated that heterozygous *Hfe* gene deletion in our mouse model of NAFLD leads to impaired glucose homeostasis in the fasted state, characterized by raised serum glucose concentrations and HOMA‐IR scores. Despite this, the dysregulation of hepatic lipid metabolism and histological evidence of increased liver injury seen previously in *Hfe* ^−/−^ mice were not seen in *Hfe* ^+/−^ mice.

Increased serum glucose in *Hfe* ^+/−^ mice is a notable finding and is significant in relation to HFE's role in insulin sensitivity, type II diabetes mellitus, and NAFLD (Nelson et al. 2012; Rong et al. 2012). This study, however, was not primarily designed to investigate glucose homeostasis. Undoubtedly, dynamic measures of glucose homeostasis such as glucose and pyruvate tolerance testing with hyperinsulinemic–euglycemic clamp studies would help future studies to characterize the extent and specific location of insulin resistance in this model.

The links between iron, insulin resistance, and type II diabetes have been extensively studied (Simcox and McClain 2013). Two large longitudinal cohort studies have demonstrated increased risk of diabetes associated with hyperferritinemia (Ford and Cogswell 1999; Montonen et al. 2012). These associations remained valid even after accounting for known confounders such as inflammation. Furthermore, therapeutic phlebotomy as a method of iron depletion has been shown in small studies to improve glycemic control in nondiabetic, prediabetic, and diabetic subjects (Facchini 1998; Fernandez‐Real et al. 2002; Houschyar et al. 2012).

Somewhat counterintuitively, previous studies have suggested enhanced insulin sensitivity in homozygous *Hfe* null mice (Huang et al. 2007, 2011). This has been proposed to be due to a lack of internalization of adipocyte ferroportin, subsequent adipocyte iron depletion, and upregulation of the expression of the insulin sensitizing adipokine, adiponectin (Gabrielsen et al. 2012). This effect, however, was not seen in our model, as heterozygous *Hfe* ^+/−^ deletion did not appear to be sufficient to interfere with serum adiponectin levels.

The findings of our study are relevant if one considers the human data regarding *Hfe* mutations and type II diabetes mellitus risk. A large meta‐analysis that reviewed studies describing *Hfe* gene polymorphisms and the risk of type II diabetes concluded that the heterozygous H63D mutation was associated with increased risk of diabetes (Rong et al. 2012). In the context of this human data, our study provides a clear framework and suitable model for future animal studies exploring the mechanistic links between HFE and type II diabetes.

Among individuals with NAFLD, the presence of heterozygous H63D mutation has been associated with higher steatosis grades and NAS score (Nelson et al. 2012). Given that H63D has less iron loading potential than the C282Y mutation, this raises the intriguing possibility of an iron‐independent role for the HFE protein in macronutrient metabolism that might protect against diabetes and NASH.

Heterozygous *Hfe* deletion did not appear to influence liver injury in this model, but it may be possible that histological differences between *Hfe* ^+/−^ and WT mice would only become evident in older mice with a more prolonged exposure to HCD and greater hepatic iron loading. Given the lack of lobular inflammation seen on liver histology, inflammatory pathways have not been explored in this model. Although it is possible that such pathways may be early markers of liver injury, analysis of these pathways would be better suited to a model with more prolonged exposure to HCD.

An interesting finding in this study was the effect of both diet and genotype on hepatic iron concentration (HIC). Increased hepatic iron in *Hfe* ^+/−^ mice is consistent with reduced stimulation of hepcidin–ferroportin axis in the setting of HFE deficiency. Humans with heterozygous C282Y mutations have been shown to have increased levels of serum ferritin, a marker of tissue iron stores, when compared to wild‐type controls (Allen et al. 2008). The lower HIC observed with HCD is consistent with other previous studies (Tan et al. 2011; Sonnweber et al. 2012). However, the lack of dietary effect on *Hamp1* mRNA when normalized to HIC suggests that hepcidin response remains appropriate in this model. Similarly, we found no dietary effect on the mRNA levels of two other regulators of iron absorption, the enterocyte iron transporter DMT1 and the ferroxidase, hephaestin, despite a previous report of dysregulation of the mRNA transcripts of these genes in response to a high‐fat diet (Sonnweber et al. 2012).

In conclusion, we have demonstrated impaired glucose and iron homeostasis with heterozygous *Hfe* gene deletion in a mouse model of NAFLD. The genetic defect, however, was not associated with increased liver injury or impaired hepatic lipid metabolism.

## Conflict of Interest

None declared.
